# Epidermal keratinocyte-specific STAT3 deficiency aggravated atopic dermatitis-like skin inflammation in mice through TSLP upregulation

**DOI:** 10.3389/fimmu.2023.1273182

**Published:** 2023-11-20

**Authors:** Zhao-Yuan Wang, Yu-Xin Zheng, Fan Xu, Ying-Zhe Cui, Xue-Yan Chen, Si-Qi Chen, Bing-Xi Yan, Yuan Zhou, Min Zheng, Xiao-Yong Man

**Affiliations:** Department of Dermatology, Second Affiliated Hospital, Zhejiang University School of Medicine, Hangzhou, China

**Keywords:** atopic dermatitis, DNCB, keratinocytes, stat3, TSLP

## Abstract

Atopic dermatitis (AD) is one of the most common inflammatory skin diseases with complex pathogenesis involving epidermal barrier dysfunction, skin microbiome abnormalities and type-2-skewed immune dysregulation. Signal transducer and activator of transcription 3 (STAT3) is a transcription factor that plays critical roles in various biological processes. However, the role of STAT3 in epidermal keratinocytes in AD remains unclear. In this study, we generated an epidermal keratinocyte-specific *Stat3*-deficient mouse strain (termed *Stat3* cKO mice). After topical 2,4-dinitrochlorobenzene (DNCB) treatment, *Stat3* cKO mice developed worsened AD-like skin inflammation with increased Ki67^+^ cells, decreased filaggrin and loricrin expression, and downregulated S100A9 and LL37. The dominant microbial population in *Stat3* cKO mice changed from *Ralstonia* to *Staphylococcus*. DNCB-treated *Stat3* cKO mice displayed more infiltrating type-2 inflammatory cells, including mast cells, eosinophils, and CD4^+^T cells, accompanied by increased skin IL-4 and serum IgE levels. Moreover, thymic stromal lymphopoietin (TSLP), mainly produced by keratinocytes, was highly expressed in the ear skin of *Stat3* cKO mice and chemoattracted more TSLPR^+^ cells. TSLP blockade significantly alleviated DNCB-induced AD-like skin inflammation in *Stat3* cKO mice. Thus, epidermal keratinocyte-specific STAT3 deficiency can aggravate AD-like skin inflammation in mice, possibly through TSLP dysregulation.

## Introduction

Atopic dermatitis (AD) is one of the most common chronic inflammatory skin diseases (1), affecting 2.7% to 20.1% of children and 2.1% to 4.9% of adults across different countries (2, 3). AD is characterized by recurrent eczematous lesions and intense itching, and has a high heterogeneity in its natural course. The onset of AD occurs early in life in approximately 80% of cases, with the remainder developing AD during adulthood (4).

The causes of AD are complex and multifactorial, including epidermal barrier defects, skin microbiome abnormalities, and type-2-skewed immune dysregulation (5). These pathophysiological processes can promote and interact with others. Disruption of skin barrier function due to filaggrin deficiency and microbial dysbiosis promotes the release of epidermal alarmins, such as interleukin (IL)-33 and thymic stromal lymphopoietin (TSLP) (6, 7); these proinflammatory signals recruit and activate type 2 inflammatory cells (8, 9); local type 2 immune responses further impair the barrier function, thereby facilitating *Staphylococcus aureus* (*S. aureus*) colonization or infection (5).

Signal transducer and activator of transcription 3 (STAT3) is the downstream transcription factor of multiple cytokines and Janus kinases (JAKs), which is involved in the regulation of immune response, and cell growth, differentiation and apoptosis (10). Previous studies have focused on the role of STAT3 signaling in immune cells. In T cells, STAT3 acts as a critical transcription factor for Th17 cell differentiation (11). In B cells, impaired STAT3 signaling can reduce their cellular and molecular maturation, which results in the production of low-affinity IgE (12). However, given that epidermal keratinocytes are not only the first line of defense against external invaders but also essential for the initiation, progression, and persistence of AD (13), the contribution of keratinocyte STAT3 to AD remains unclear.

Therefore, in this study, we evaluated the role of STAT3 in keratinocytes by generating mice with inducible keratinocyte-specific deletion of STAT3 and treating them with 2,4-dinitrochlorobenzene (DNCB) to induce AD-like skin inflammation. Mice with keratinocyte-specific STAT3 deficiency (termed *Stat3* cKO mice) developed more severe DNCB-induced AD-like skin inflammation accompanied by skin barrier impairment, *Staphylococcus*-dominant microbial dysbiosis, more infiltrating type-2 inflammatory cells, and increased IL-4 levels. Moreover, TSLP expression was increased in *Stat3* cKO mice, which recruited more target cells to the affected skin. TSLP blockade attenuated DNCB-induced AD-like skin inflammation in *Stat3* cKO mice, suggesting that keratinocyte-specific STAT3 dysfunction leads to TSLP-mediated skin inflammation in AD.

## Materials and methods

### Generation of mice with keratinocyte-specific deletion of Stat3

To generate keratinocyte-specific *Stat3* cKO mice, *Stat3^f/f^* mice (B6.129S1-*Stat3^tm1Xyfu^*/J mice, Jackson Lab #016923) were crossed with *K14-CreERT*^+^ mice (STOCK Tg(KRT14-cre/ERT)20Efu/J mice, Jackson Lab # 005107), both of which were purchased from Jackson Laboratory. To confirm the deficiency of STAT3 in the mouse epidermis induced by intraperitoneal injection of tamoxifen, Western blotting and immunohistochemistry were performed using STAT3 and phospho-STAT3 specific antibodies. All mouse experiments were performed under a protocol approved by the Second Affiliated Hospital Zhejiang University School of Medicine Animal Care Committee.

### Establishment of AD-like skin lesions

AD-like skin lesions were induced by topical application of DNCB (Sigma-Aldrich, St. Louis, MO, USA), with minor modifications to the protocol. Briefly, the ear skin of mice was sensitized once on Day -7 by applying 20 μL of 1% DNCB dissolved in acetone: olive oil (3:1). Beginning on Day 0, mice were challenged by applying 20 μL of 0.5% DNCB to the ears every other day for up to 21 days. The thickness of ear skin was measured with a digital caliper once a week.

### Histological analysis

Ear tissues were fixed with 10% formalin and embedded in paraffin. Afterwards, paraffin-embedded sections (4 μm) were cut, deparaffined and stained with hematoxylin-eosin (H&E) or toluidine blue for light microscopic examinations.

### Immunohistochemistry and immunofluorescence

Formalin-fixed, paraffin-embedded tissues were subjected to sectioning, deparaffinization, and rehydration. Then, heat-induced epitope retrieval was performed in a 0.01M sodium citrate buffer (pH 6.0), followed by blocking with normal goat serum (Boster, Beijing, China) for 1 hour at room temperature.

For IHC, tissue sections were incubated with p-STAT3 (Cell Signaling Technology, 9145, 1:250) and Ki67 (Abcam, ab264429, 1:500) antibodies respectively at 4°C overnight. Subsequently, HRP-labeled anti-rabbit secondary antibodies (Boster, SV0002, 1:500) were applied with DAB high-sensitivity substrate chromogen solution (Vector Laboratories, Burlingame, CA, USA).

For IF, slides were incubated with K14 (Abcam, ab181595, 1:500), CD4 (Abcam, ab183685, 1:500), CD8 (R&D, MAB116, 1:100), loricrin (Proteintech, 55439-1-AP, 1:100), filaggrin (Abcam, ab81468, 1:200), S100A9 (Abcam, ab63818, 1:250), LL37 (Abcam, ab180760, 1:250), TSLP (Abcam, ab188766, 1:250), CD68 (Santa Cruz, sc-17832, 1:100), CD11c (Abcam, ab11029, 1:200), TSLPR (BioLegend, 151802, 1:200), respectively at 4°C overnight. Then, Alexa 488 or Alexa 555 conjugated secondary antibodies (Thermo Fisher Scientific, 1:200) plus DAPI (Roche, Mannheim, Germany) were used for visualization.

### Measurement of total serum IgE by ELISA

To detect the total serum IgE after DNCB modeling, blood samples were collected from the retroorbital plexus on Day 21 followed by centrifugation at 3000 rpm for 10 min at 4°C. The total serum IgE was measured by using the mouse ELISA kit (Mouse IgE ELISA MAX Deluxe, BioLegend).

### Flow cytometric analysis

After separating the epidermis and dermis of mouse ear skin, the epidermis was digested with 0.25% trypsin (Thermo Fisher Scientific, USA), whereas the dermis was digested with 1 mg/ml collagenase (Sigma-Aldrich, St. Louis, MO). After neutralization with fetal bovine serum, the mixture was centrifuged at 1000 rpm for 5 min, and the cells were resuspended in PBS to obtain a single-cell suspension. After Fc receptor blocking with Mouse TruStain FcX PLUS (1:100, BioLegend, San Diego, CA), the cells were incubated with Live/Dead Zombie dye (UV, BioLegend, 423106, 1:100) and antibodies specific for CD45 (BV510, BioLegend, 103138, 1:100) and FcϵRIα (PE/Cyanine7, BioLegend, 134318, 1:100) in the dark at room temperature for 30 min. After centrifugation, the cells were suspended in PBS and detected by the Beckman CytoFlex LX flow cytometer using CytExpert software (Beckman Coulter, Brea, CA).

### Preparation of tissue homogenate

After sacrifice, the mouse ear was removed and cut into pieces. Then, the tissues were grinded to powder in mortar and lysed with RIPA buffer containing phosphatase and protease inhibitors. The total protein concentration of the homogenate was measured by the Pierce BCA Protein Assay Kit (Thermo Fisher Scientific, 23227) and further diluted to 5 μg/μl. These samples were stored at -80°C for subsequent cytokine assays.

### Measurement of cytokines by a Luminex assay

For the simultaneous detection of various cytokines in the mouse ear skin after DNCB induction, the R&D Luminex Assay (Catalog Number LXSAMSM) was used according to the manufacturer’s instructions. Here, IL-4, IL-10, IFN-γ, TNF-α, IL-1β, and IL-6 were measured, performed by the Novogene Bioinformatics Technology Co. (Beijing, China).

### Full-length 16s ribosomal RNA sequencing and data analysis

To profile the skin microbiota after DNCB treatment, the mouse ear skin was swabbed with sterile swabs which were further snap-frozen on dry ice and sent out for full-length 16s ribosomal RNA sequencing using Pacific Bioscience platform. After obtaining clean reads, the program Uparse V7.0.1001 was used to cluster these sequences into Operational Taxonomic Units with an average percent identity of 97%. Based on the SILVA database, the software Mothur was used for taxonomic classification. The above full-length 16s rRNA sequencing and bioinformatics analysis were performed by the Novogene Bioinformatics Technology Co. (Beijing, China).

### RNA sequencing and bioinformatics analysis

RNA sequencing was performed on the total epidermal RNA from mouse ear skin after being treated with DNCB or vehicle on 21 days. Differentially expression analysis was conducted by the DESeq2 R package (1.20.0). The *P*-values were adjusted using the Benjamini & Hochberg method. Adjusted *P* < 0.05 and |log2 fold change| > 2 were set as the threshold to define statistical significance. Data processing and analysis are detailed in **Supplementary Materials**, which were performed by the Novogene Bioinformatics Technology Co. (Beijing, China).

### Measurement of TSLP and TSLP blockade

For the detection of TSLP in the ear skin, the mouse ELISA kit (Mouse TSLP ELISA MAX Deluxe, BioLegend) was used according to the manufacturer’s protocol. To block TSLP activity in the skin, DNCB-treated *Stat3* cKO mice and *Stat3^f/f^* mice on Day 23 (D0) were injected at ears with 20 μg of neutralizing TSLP antibody (BioLegend, 515202) or IgG2a control antibody (BioLegend, 407102). One hour after injection, both mice received topical DNCB treatment. The above procedures were carried out every other day until the 8^th^ day, as well as the measurement of the ear thickness and dermatitis score.

### Statistical analysis

The experimental data were analyzed by Prism 8.0 Software (GraphPad Software, La Jolla, CA, USA) and significance was assessed by the two-way ANOVA analysis or Mann-Whitney U test. For all experiments with error bars, standard deviations (SD) were calculated to indicate variations, and the data are represented as the Mean ± SD. Here, *P* < 0.05 was considered significant (*P < 0.05, **P < 0.01, ***P < 0.001, ****P < 0.0001).

## Results

### Keratinocyte-specific STAT3 deficiency exacerbated DNCB-induced AD-like skin inflammation

To explore the role of epidermal keratinocyte STAT3 in the pathogenesis of AD, we developed tamoxifen-inducible *K14-CreERT^+^Stat3^flox/flox^* mice (termed *K14-CreERT^+^-Stat3^f/f^* mice). Intraperitoneal injection of tamoxifen into *K14-CreERT^+^-Stat3^f/f^* mice for 5 consecutive days lead to epidermal keratinocyte-specific STAT3 deficiency (termed *Stat3* cKO) (**Supplementary Figure S1A**). Taking *K14-CreER^-^Stat3^flox/flox^* mice (termed *Stat3^f/f^* mice) as the control, the efficacy of STAT3 deficiency in the epidermis was confirmed by Western blotting and immunohistochemistry (IHC) (**Supplementary Figures S1B, C**).

**Figure 1:**
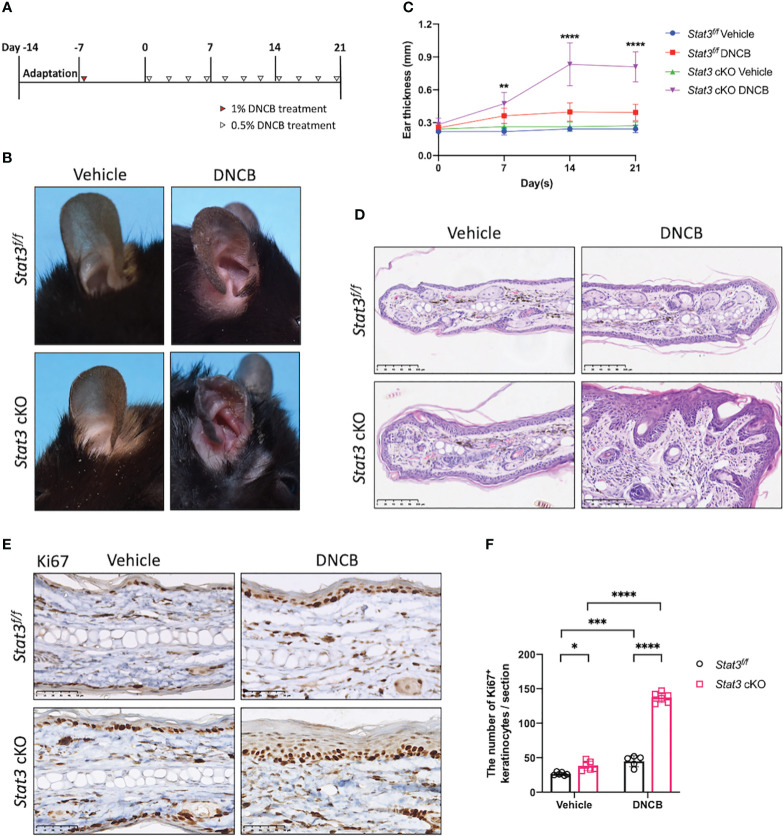
STAT3 deficiency in basal keratinocytes exacerbated DNCB-induced AD-like skin inflammation. **(A)** Schematic diagram of the experimental design of DNCB-induced AD-like skin inflammation. **(B, D)** Representative phenotypes and histological changes in *Stat3^f/f^* mice and *Stat3* cKO mice after DNCB or vehicle treatment on Day 21. Bar = 250 μm. **(C)** During the induction period, ear thickness (mm) was measured weekly (n ≥ 10). **(E)** Representative IHC staining of Ki67 and **(F)** Ki67-positive keratinocyte counts (n = 5-6). Bar = 50 μm. The data are presented as the mean ± SD, *P < 0.05, **P < 0.01, ***P < 0.001, and ****P < 0.0001.

After tamoxifen induction, DNCB was topically applied to the ears of the mice to simulate AD-like skin inflammation (**Figure 1A**). DNCB is a chemical hapten commonly used to induce allergic contact dermatitis and thought to evoke a Th1-dominated immune response. However, repeated challenges with DNCB over an extended period cause a chronic Th2-dominated inflammatory response similar to that in human AD (14). On day 21, the DNCB-treated ears of *Stat3* cKO mice were red, thickened, and swollen, accompanied by exudation and crusting. Interestingly, these AD-like skin lesions were more severe than those in *Stat3^f/f^* mice (**Figure 1B**; **Supplementary Figure S2A**). The thickness of DNCB-treated ear in *Stat3* cKO mice was significantly increased from Day 7, and the difference became greater over time (**Figure 1C**). Histologically, the DNCB-treated ear skin of *Stat3* cKO mice showed marked acanthosis, increased granular layers, and thickened dermis along with dermal inflammatory cell infiltration (**Figure 1D**), which were similar to the histological changes observed in human AD. In addition, *Stat3* cKO mice exhibited a significant increase in the number of Ki67^+^ cells among keratinocytes, which is a marker of epidermal hyperplasia (**Figures 1E, F**). After DNCB treatment, the phosphorylation of STAT3 was confirmed using IHC staining. As expected, keratinocyte-specific deficiency of phosphorylated STAT3 was observed in *Stat3* cKO mice. Besides, there was increased STAT3 activation in *Stat3^f/f^* mice after DNCB treatment (**Supplementary Figure S2B**). Collectively, these results indicate that keratinocyte-specific STAT3 dysfunction exacerbated DNCB-induced AD-like skin inflammation.

### Dysregulation of epidermal barrier, antimicrobial peptides and microbiome in Stat3 cKO mice with AD-like skin inflammation

**Figure 2:**
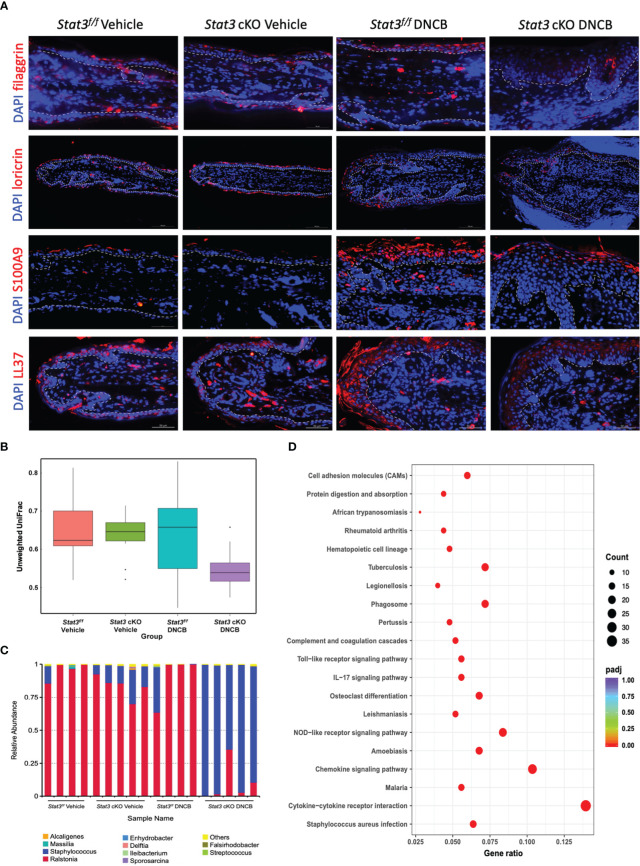
Epidermal barrier dysfunction was aggravated in *Stat3* cKO mice with AD-like skin inflammation, accompanied by AMP insufficiency and microbial dysbiosis. **(A)** Representative IF staining of filaggrin, loricrin, S100A9, and LL37 (red) in the ear skin of *Stat3^f/f^* mice and *Stat3* cKO mice after DNCB or vehicle treatment. Bar = 50 μm. **(B, C)** The microbial diversity on the surface of the ear skin and the relative abundance of different microbial genera determined by full-length 16s ribosomal RNA sequencing (n = 4-5). **(D)** KEGG pathway analysis of differentially expressed genes identified by RNA sequencing between the epidermis of *Stat3^f/f^* mice and *Stat3* cKO mice after DNCB treatment (n = 3-4, adjusted *P* < 0.05, |log2-fold change| > 2).

The epidermis is the first line of defense between the host and its environment. In AD, there usually exists a defective epidermal barrier, whose critical importance in the disease development has been apparent. To determine whether STAT3 dysfunction may impair skin barrier function, the expression of filaggrin and loricrin was studied by using immunofluorescence (IF). In vehicle-treated ear skin, there was relatively continuous expression of filaggrin and loricrin (**Figure 2A**). However, after repeated stimulation with DNCB, both were downregulated, especially in *Stat3* cKO mice, in which they were barely expressed (**Figure 2A**).

In addition to the above structural proteins, keratinocytes also synthesize various host defense molecules such as antimicrobial peptides (AMPs), which exert broad-spectrum effects against bacteria, fungi, and viruses (15). Thus, we examined the expression of S100A9 and cathelicidin (LL37) in the ear skin by IF. As shown in **Figure 2A**, S100A9 was barely expressed in *Stat3^f/f^* mice that received topical application of vehicle, while LL37 was slightly expressed in the cytoplasm of keratinocytes in the upper epidermis. After topical DNCB treatment, the expression of S100A9 and LL37 in *Stat3^f/f^* and *Stat3* cKO mice was upregulated, and these proteins were mainly distributed in the upper layers of the ear epidermis. However, compared with *Stat3^f/f^* mice, *Stat3* cKO mice showed a reduced expression level of both proteins (**Figure 2A**).

AD flares are generally associated with a disordered microbiome, with *S. aureus* being a dominant colonizer and pathogen (16). Dysfunction of the epidermal barrier, together with an insufficient upregulation of specific AMPs, might further enhance *S. aureus* colonization (1). Therefore, we performed full-length 16s ribosomal RNA sequencing to identify microbial populations on the ear skin surface. The microbial diversity of DNCB-treated *Stat3* cKO mice was decreased compared with that of the other groups (**Figure 2B**). Moreover, the relative abundance of genera was different among the 4 groups (**Figure 2C**). On the ear skin of *Stat3^f/f^* mice, the dominant genus was *Ralstonia* regardless of topical vehicle or DNCB treatment. However, in *Stat3* cKO mice, the *Staphylococcus* genus with a small proportion appeared on the vehicle-treated ear skin, and became dominant after DNCB treatment (**Figure 2C**). To further explore the effects of *Staphylococcus* dominance, we analyzed the epidermal transcriptome using RNA sequencing, which identified 651 upregulated genes and 139 downregulated genes (adjusted *P* < 0.05, |log2-fold change| > 2, **Supplementary Figure S3A**). KEGG pathway analysis showed that the upregulated genes were most significantly enriched in the *S. aureus* infection pathway, suggesting that *Staphylococcus* dominance influenced the expression of related genes in *Stat3* cKO mice (**Figure 2D**; **Supplementary Figure S3B**).

Taken together, these data suggest that epidermal barrier impairment was aggravated in *Stat3* cKO mice with AD-like skin inflammation, accompanied by specific AMP insufficiency and *Staphylococcus*-dominant microbial dysbiosis.

### Activation of type-2 inflammation in the DNCB-treated ear skin of Stat3 cKO mice

**Figure 3:**
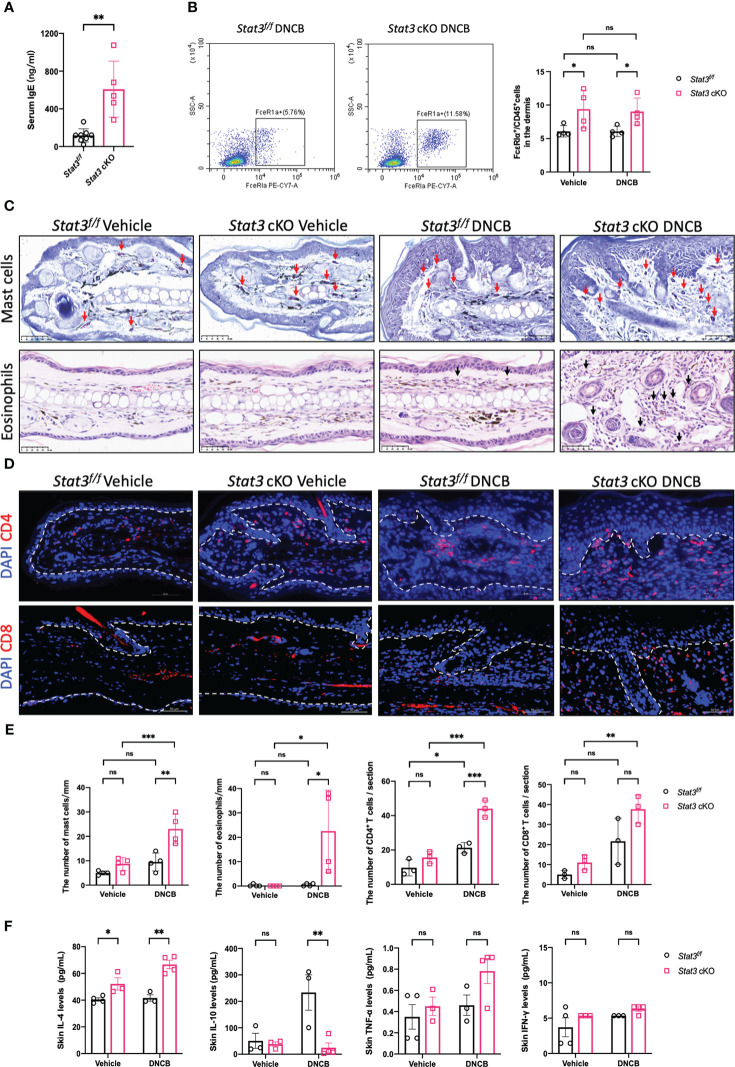
Keratinocyte-specific STAT3 deficiency predominantly increased the infiltration of type-2 inflammatory cells and the secretion of related cytokines. **(A)** Analysis of the serum IgE levels (ng/ml) in *Stat3^f/f^* mice (n = 8) and *Stat3* cKO mice (n = 5) after DNCB treatment using ELISA. **(B)** Flow cytometry analysis of FcϵRIα^+^ cells in the dermis of *Stat3^f/f^* mice and *Stat3* cKO mice (n = 4). **(C)** Toluidine blue staining of mast cells (red arrows) and H&E staining of eosinophils (black arrows) in mouse ear skin of each group. Bar = 50 μm. **(D)** Representative IF staining of CD4^+^ or CD8^+^ cells (red) in the ear skin of *Stat3^f/f^* mice and *Stat3* cKO mice. Bar = 50 μm. **(E)** Statistical analysis results of the number of mast cells, eosinophils, and CD4^+^ or CD8^+^ cells (n = 3-4). **(F)** The expression levels of Il-4, IL-10, TNF-α, and IFN-γ (pg/ml) in the ear skin of *Stat3^f/f^* mice and *Stat3* cKO mice (n = 3-4). The data are presented as the mean ± SD; *P* > 0.05 denotes not significant (ns); *P < 0.05, **P < 0.01, and ***P < 0.001.

Since the cutaneous inflammation of AD is characterized by sequential and progressive patterns of inflammatory cell infiltration, predominantly involving type-2-skewed immune dysregulation, we studied several type-2 inflammatory cells and cytokines. Serum IgE levels were significantly elevated in *Stat3* cKO mice compared with *Stat3^f/f^* mice after DNCB treatment (**Figure 3A**). As a high-affinity Fc receptor for IgE, FcϵRIα is abundantly expressed on mast cells and basophils (17). Using flow cytometry, we found that the proportion of FcϵRIα^+^ cells in the ear dermis of *Stat3* cKO mice was significantly increased (**Figure 3B**). Among these FcϵRIα^+^ cells, mast cells were detected by toluidine blue staining. *Stat3* cKO mice treated with DNCB exhibited more infiltrating mast cells in the dermis (**Figures 3C, E**). The presence of eosinophils in AD inflammation has long been established (18). Consistently, there was a significant increase in eosinophil number in *Stat3* cKO mice with DNCB-induced AD-like skin inflammation (**Figures 3C, E**).

In addition to the above inflammatory cells, T-cell infiltration is crucial for the development of AD skin inflammation. Using IF, we found that the number of CD4^+^ T cells was significantly increased in the DNCB-treated ear skin of both mice, but *Stat3* cKO mice exhibited more CD4^+^ T cells, especially in the dermal-epidermal interface (**Figures 3D, E**). Unlike CD4^+^ T cells, there were no significant changes in the number of CD8^+^ T cells (**Figures 3D, E**).

We further measured the expression levels of cytokines in the ear skin using a Luminex Assay. Compared with *Stat3^f/f^* mice, the type-2 cytokine IL-4 was significantly upregulated in *Stat3* cKO mice, regardless of vehicle or DNCB treatment (**Figure 3F**). In contrast, the inhibitory cytokine IL-10 expression was significantly decreased in the DNCB-treated ear skin of *Stat3* cKO mice (**Figure 3F**). In addition, the expression of the proinflammatory cytokine TNF-α and type-1 cytokine IFN-γ showed no significant changes (**Figure 3F**).

Collectively, these results indicate that STAT3 dysfunction in basal keratinocytes predominantly promoted the infiltration of type-2 inflammatory cells and the secretion of related cytokines.

### Keratinocyte-specific STAT3 deficiency promoted the production of TSLP by keratinocytes

**Figure 4:**
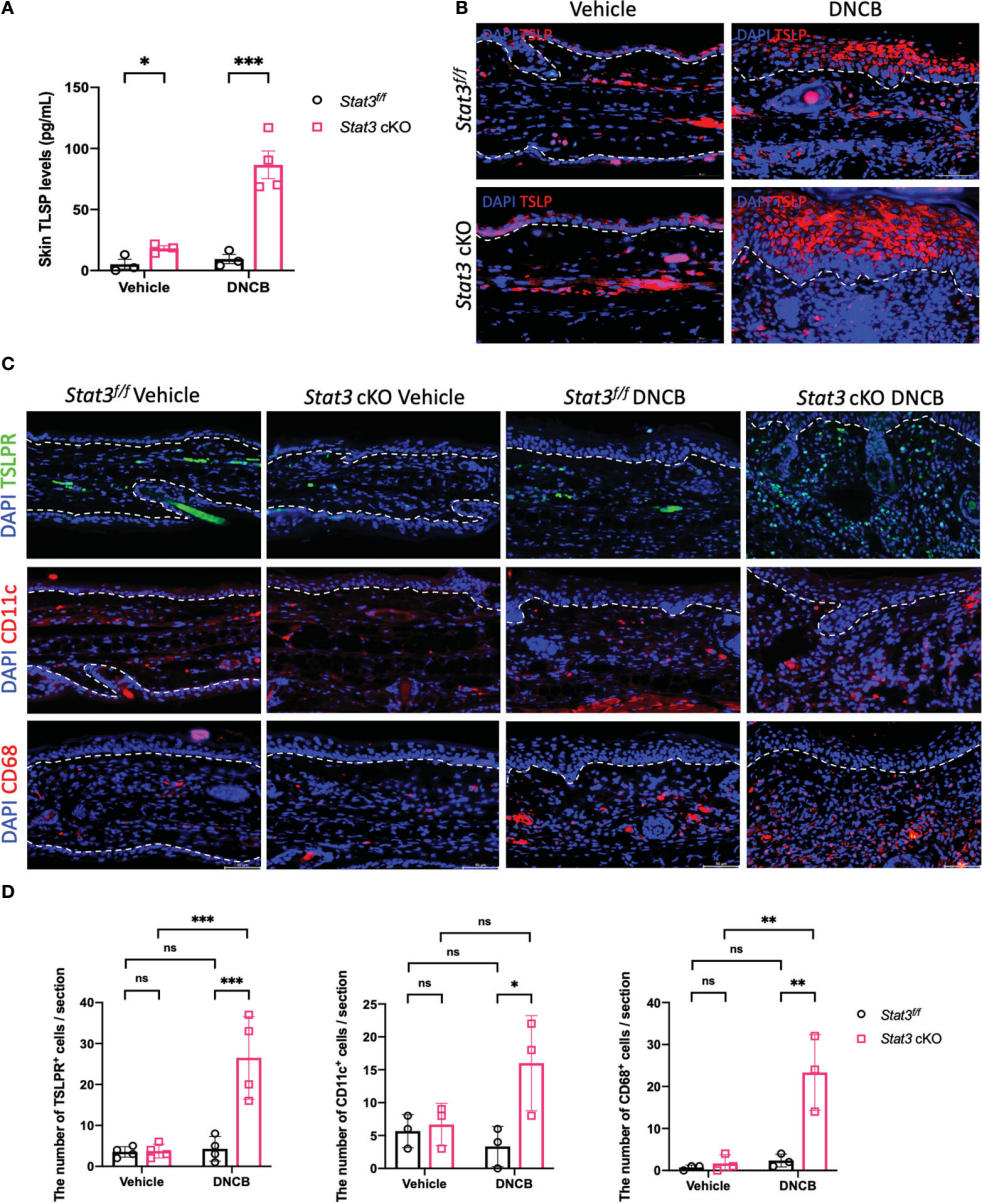
The production of TSLP by keratinocytes was increased in *Stat3* cKO mice, which recruited more target cells in AD-like skin inflammation. **(A)** Analysis of TSLP expression level (pg/ml) in the ear skin of *Stat3^f/f^* mice and *Stat3* cKO mice after DNCB or vehicle induction by ELISA (n = 3-4). **(B)** IF staining showed that TSLP was primarily expressed in keratinocytes (red). Bar = 50 μm. **(C)** IF staining revealed that the infiltration of TSLPR^+^ cells (green), CD11c^+^ cells (red), and CD68^+^ cells (red) was increased in the dermis of DNCB-treated *Stat3* cKO mice compared with *Stat3^f/f^* mice. **(D)** Statistical analysis of the abovementioned cells (n = 3-4). The data are presented as the mean ± SD; *P* > 0.05 denotes ns; *P < 0.05, **P < 0.01, and ***P < 0.001.

In barrier-impaired skin, keratinocytes send proinflammatory signals through alarmins including thymic stromal lymphopoietin (TSLP) (5). TSLP serves as a key “bridge”, which exerts its biological effects by binding to a high-affinity heteromeric complex composed of a TSLP receptor (TSLPR) subunit and a IL-7 receptor (IL-7R) α chain (19). This functional TSLPR is expressed by a variety of immune cell populations, including dendritic cells (DCs), monocytes/macrophages, mast cells, eosinophils, basophils, and T and B cells (20, 21). Our ELISA data showed that the expression level of TSLP was significantly increased in the ear skin of *Stat3* cKO mice, regardless of vehicle or DNCB induction (**Figure 4A**). IF staining revealed that TSLP was primarily expressed in keratinocytes, and the expression differences among these groups were consistent with those revealed by ELISA (**Figure 4B**). Moreover, there exhibited a significantly increased infiltration of TSLPR^+^ cells in the ear dermis of DNCB-treated *Stat3* cKO mice compared with that of DNCB-treated *Stat3^f/f^* mice (**Figures 4C, D**). Specifically, there was a significant increase in the number of infiltrating CD11c^+^ DCs and CD68^+^ macrophages into the skin (**Figures 4C, D**). These data reveal that keratinocyte-specific STAT3 deficiency promoted the expression and secretion of TSLP by keratinocytes, which recruited specific target cells in AD-like skin inflammation.

### TSLP blockade alleviated DNCB-induced AD-like skin inflammation of Stat3 cKO mice

**Figure 5:**
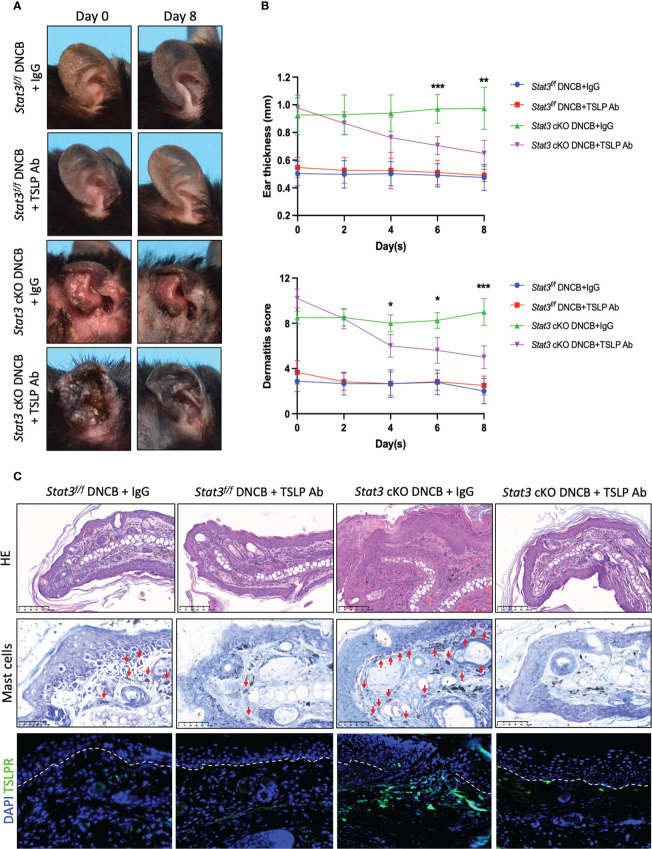
DNCB-induced AD-like skin inflammation of *Stat3* cKO mice was alleviated by an anti-TSLP neutralizing antibody. **(A)** Representative photographs of DNCB-treated *Stat3^f/f^* mice and *Stat3* cKO mice after anti-TSLP antibody or isotype IgG antibody injection on Day 8. **(B)** The ear thickness (mm) and dermatitis score were measured every other day (n = 5-8). **(C)** Analysis of the histological changes in ear skin by HE staining (Bar = 100 μm), mast cells by toluidine blue staining (red arrows, Bar = 50 μm), and TSLPR-positive cells by IF staining (green, Bar = 50 μm). The data are presented as the mean ± SD; *P* > 0.05 denotes ns; *P < 0.05, **P < 0.01, and ***P < 0.001.

Several studies have reported that keratinocyte-derived cytokine TSLP acts as a master switch of AD skin inflammation (22, 23). To determine whether the exacerbation of AD-like skin inflammation was triggered by TSLP, the ear skin of *Stat3* cKO mice was injected with an anti-TSLP neutralizing antibody during DNCB challenge. We found that anti-TSLP antibody treatment attenuated DNCB-induced AD-like skin inflammation in *Stat3* cKO mice (**Figure 5A**), as the ear thickness and dermatitis score were significantly decreased (**Figure 5B**). Histologically, the ear skin of *Stat3* cKO mice injected with the anti-TSLP antibody showed decreased epidermal and dermal thickness, reduced exudation and crusting, and fewer infiltrating dermal inflammatory cells, including mast cells (**Figure 5C**). Moreover, there was a significant decrease in the number of TSLPR^+^ cells in the dermis of *Stat3* cKO mice after anti-TSLP antibody treatment (**Figure 5C**). These results suggest that TSLP was the critical factor underlying severe AD-like skin inflammation in *Stat3* cKO mice.

## Discussion

STAT3 is well documented as an important regulator of complex biological functions. Increasing evidence suggests that STAT3 is involved in the development of allergic inflammation, but the role of STAT3 in epidermal keratinocytes in AD has not been directly evaluated. In this study, we found that *Stat3* cKO mice exhibited worsened AD-like skin inflammation characterized by a defective skin barrier, *Staphylococcus*-dominant microbial dysbiosis, more infiltrating immune cells, and an increased concentration of keratinocyte-derived TSLP.

Previous studies have found that the transcription of the *STAT3* gene is increased in lesional skin versus nonlesional skin in AD patients (24). Furthermore, as a downstream transcription factor of IL-4, IL-13, and IL-22, activated STAT3 in keratinocytes acts as a negative regulator of keratinocyte differentiation by suppressing the expression of filaggrin and loricrin, leading to epidermal barrier dysfunction (25, 26). On the other hand, dominant-negative mutations of the *STAT3* gene result in the classical autosomal dominant hyper-IgE syndrome (HIES) (27), which is characterized by AD, recurrent infections, and elevated serum IgE levels and eosinophil counts (28). AD and HIES have overlapping clinical features, but the changes in STAT3 expression and function are the opposite in these conditions, suggesting that STAT3 might play bidirectional roles in the development of AD skin lesions. Here, by using mice with keratinocyte-specific deletion of *STAT3*, we provide relatively direct evidence for the potential inhibitory role of keratinocyte STAT3 in the pathogenesis of AD-like skin inflammation.

TSLP is a cytokine expressed by epithelial cells, including keratinocytes (21). Recent work has highlighted the role of TSLP in various inflammatory diseases, which is an IL-7-like cytokine initiating and promoting type-2 inflammation, including AD, allergic rhinitis, and asthma (21). TSLP was found to be highly expressed in the lesional skin of AD patients and to potently activated DCs to secrete Th2-recruiting chemokines. These TSLP-activated DCs induce Th2 cell differentiation to produce IL-4, IL-5, IL-13, and TNF-α, while downregulating IL-10 and IFN-γ (29). Interestingly, these upregulated cytokines can synergistically promote the production of TSLP by keratinocytes (30), suggesting the existence of a positive feedback loop that amplifies skin inflammation. In addition, keratinocyte TSLP can trigger the atopic march in mice (8), and mice overexpressing TSLP in keratinocytes showed a spontaneous AD-like phenotype, with the development of eczematous lesions, increased circulating Th2 cells, and higher serum IgE levels (31). In our study, the expression level of TSLP was significantly increased in the skin of *Stat3* cKO mice and was further elevated after DNCB induction. More importantly, blockade of TSLP activity by TSLP neutralizing antibody markedly reduced AD-like skin inflammation in *Stat3* cKO mice. Thus, TSLP may be an important determinant for promoting AD-like skin inflammation in the absence of keratinocyte STAT3.

At barrier interfaces, TSLP expression can be triggered by environmental stimuli, including allergens, bacterial and fungal products, viruses, mechanical injury, and cigarette smoke extracts (20). In addition, several endogenous factors, such as proinflammatory cytokines, Th2-related cytokines and IgE, have positive effects on TSLP production (32). However, it is still unclear how STAT3 dysfunction promotes the expression of TSLP in keratinocytes. It has been indicated that *S. aureus*-derived ligands of TLRs can induce TSLP expression in keratinocytes, leading to Th2-skewed sensitization to environmental allergens and *S. aureus*-derived allergens, or both (33, 34). In this study, *Staphylococcus* dominance and corresponding transcriptomic alterations in the skin were found in *Stat3* cKO mice with AD-like skin inflammation, suggesting that *Staphylococcus*-dominant microbial colonization may be trigger TSLP expression in *Stat3* cKO mice. Future studies should address this important issue and clarify the molecular interactions or pathways involved in the process.

Atopic skin has been characterized by a relatively impaired induction of AMPs (35). Ong et al. firstly found that the expression of AMPs, including LL37 and human β-defensin 2 (hBD2), was significantly decreased in atopic lesions versus psoriatic lesions (35). Some studies have attributed this impairment to elevated Th2-related cytokines in AD, such as IL-4, IL-13, and TSLP (15, 36, 37). Among them, IL-4 and IL-13 negatively regulate LL-37 and hBD expression through STAT6 in keratinocytes (36, 37), while TSLP downregulates the production of S100A7 and hBD2 by keratinocytes via the JAK2/STAT3-dependent mechanism (15). Here, keratinocyte-specific STAT3 dysfunction led to increased TSLP and IL-4 levels accompanied by decreased S100A9 and LL37 levels. The detailed mechanisms regulating the expression of AMPs in keratinocytes need further investigation.

In summary, our findings provide insights into the role of keratinocyte STAT3 in regulating AD skin inflammation. Future studies elucidating the regulatory mechanisms among STAT3, TSLP, and AMPs should help identify new therapeutic strategies for AD.

## Data availability statement

The datasets presented in this study can be found in online repositories. The names of the repository/repositories and accession number(s) can be found below: SRP451094 (SRA).

## Ethics statement

The animal study was approved by the Second Affiliated Hospital Zhejiang University School of Medicine Animal Care Committee. The study was conducted in accordance with the local legislation and institutional requirements.

## Author contributions

Z-YW: Conceptualization, Formal Analysis, Funding acquisition, Methodology, Project administration, Visualization, Data curation, Investigation, Validation, Writing – original draft. Y-XZ: Conceptualization, Data curation, Investigation, Methodology, Validation, Writing – original draft. FX: Writing – original draft, Conceptualization, Data curation, Investigation. Y-ZC: Investigation, Writing – original draft, Validation. X-YC: Investigation, Writing – original draft. S-QC: Methodology, Writing – review & editing. B-XY: Writing – review & editing, Supervision. YZ: Supervision, Writing – review & editing. MZ: Supervision, Writing – review & editing. X-YM: Writing – review & editing, Conceptualization, Formal Analysis, Funding acquisition, Methodology, Project administration, Resources, Visualization.
