# Supplementary figures and images for: Epidermal keratinocyte-specific STAT3 deficiency aggravated atopic dermatitis-like skin inflammation in mice through TSLP upregulation
Source: Front Immunol. 2023 Nov 20;14:1273182. doi: 10.3389/fimmu.2023.1273182 (PMC10694200; doi:10.3389/fimmu.2023.1273182)

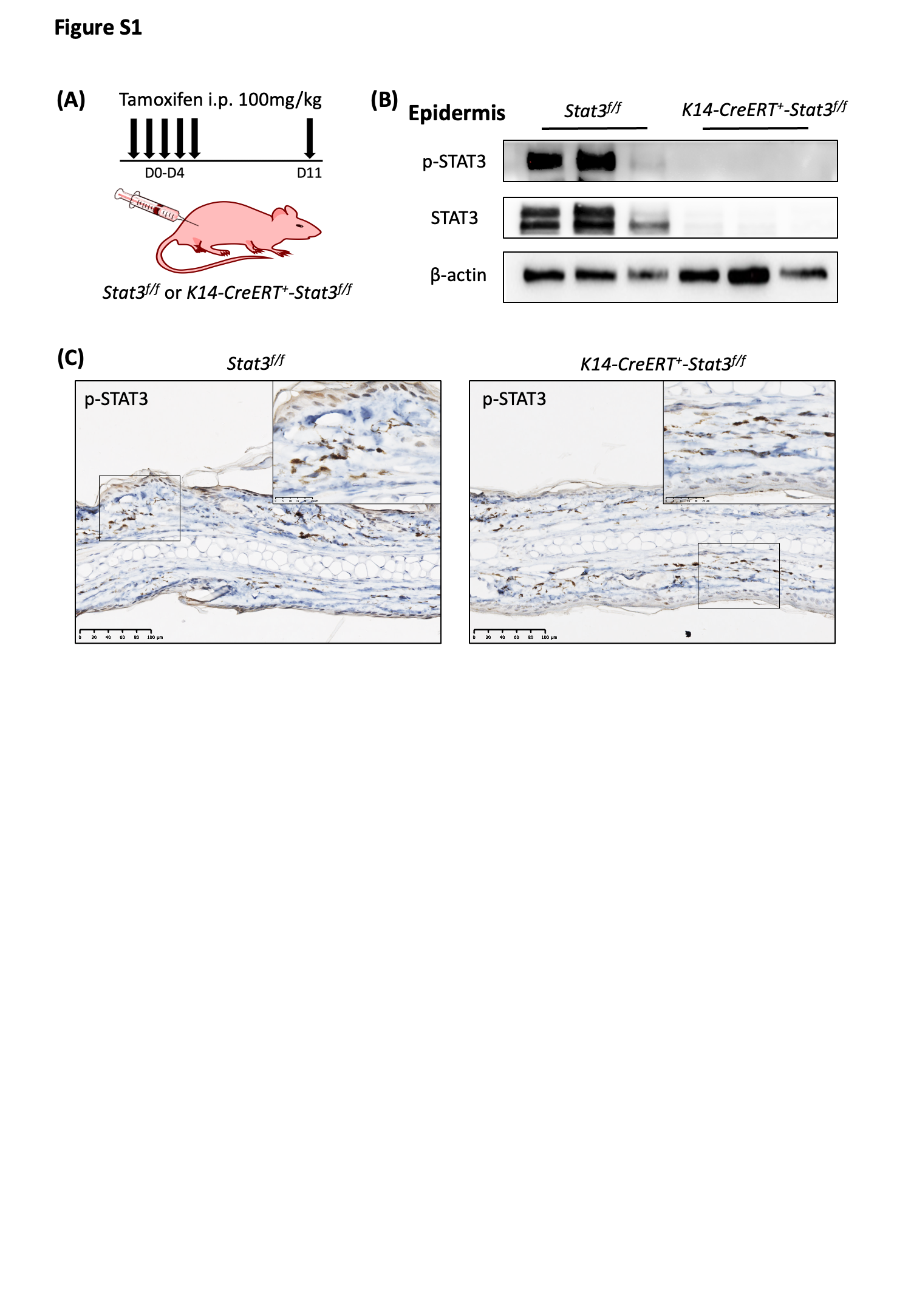

Supplement: Supplementary file 1 [file Image_1.tiff]

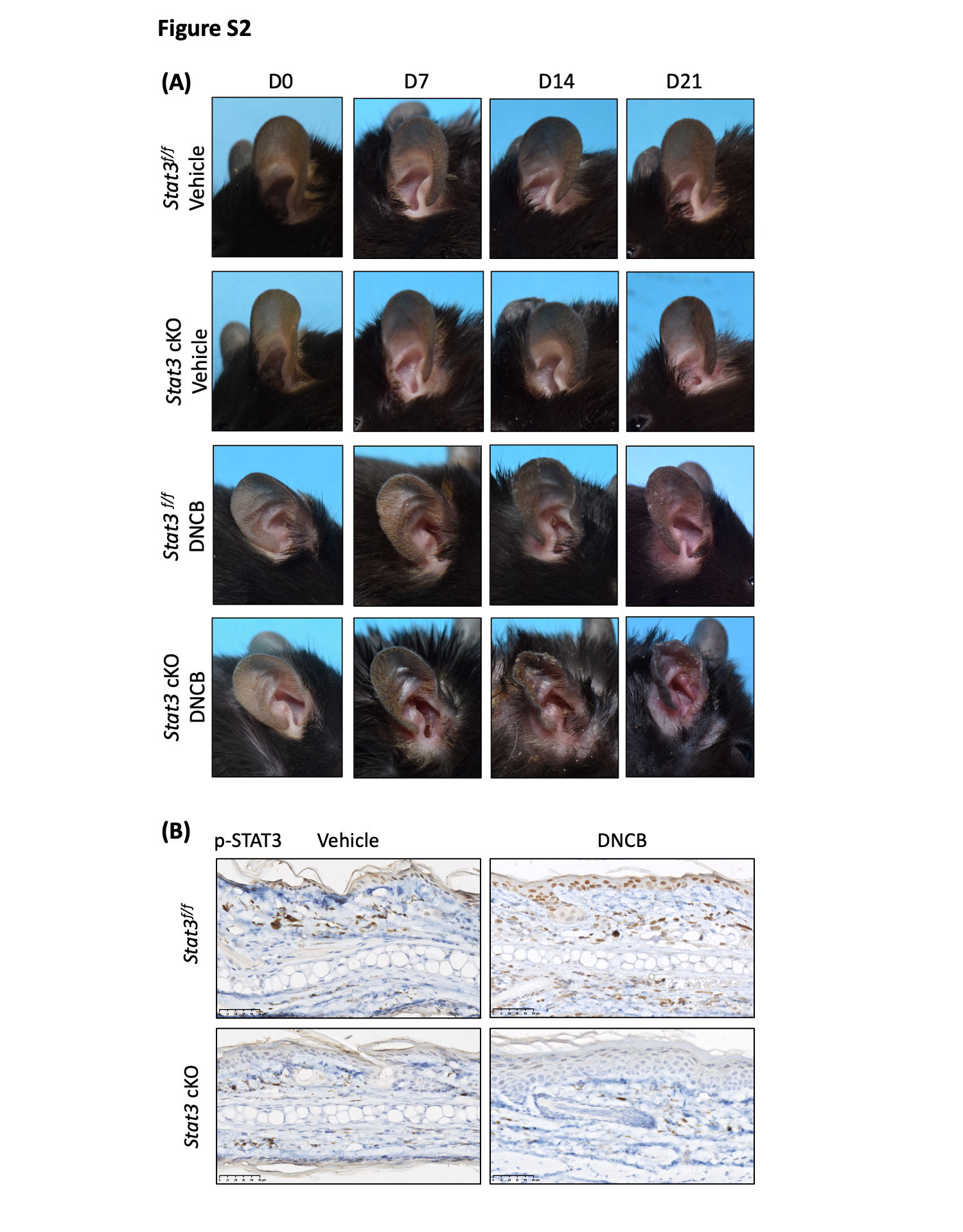

Supplement: Supplementary file 2 [file Image_2.tiff]

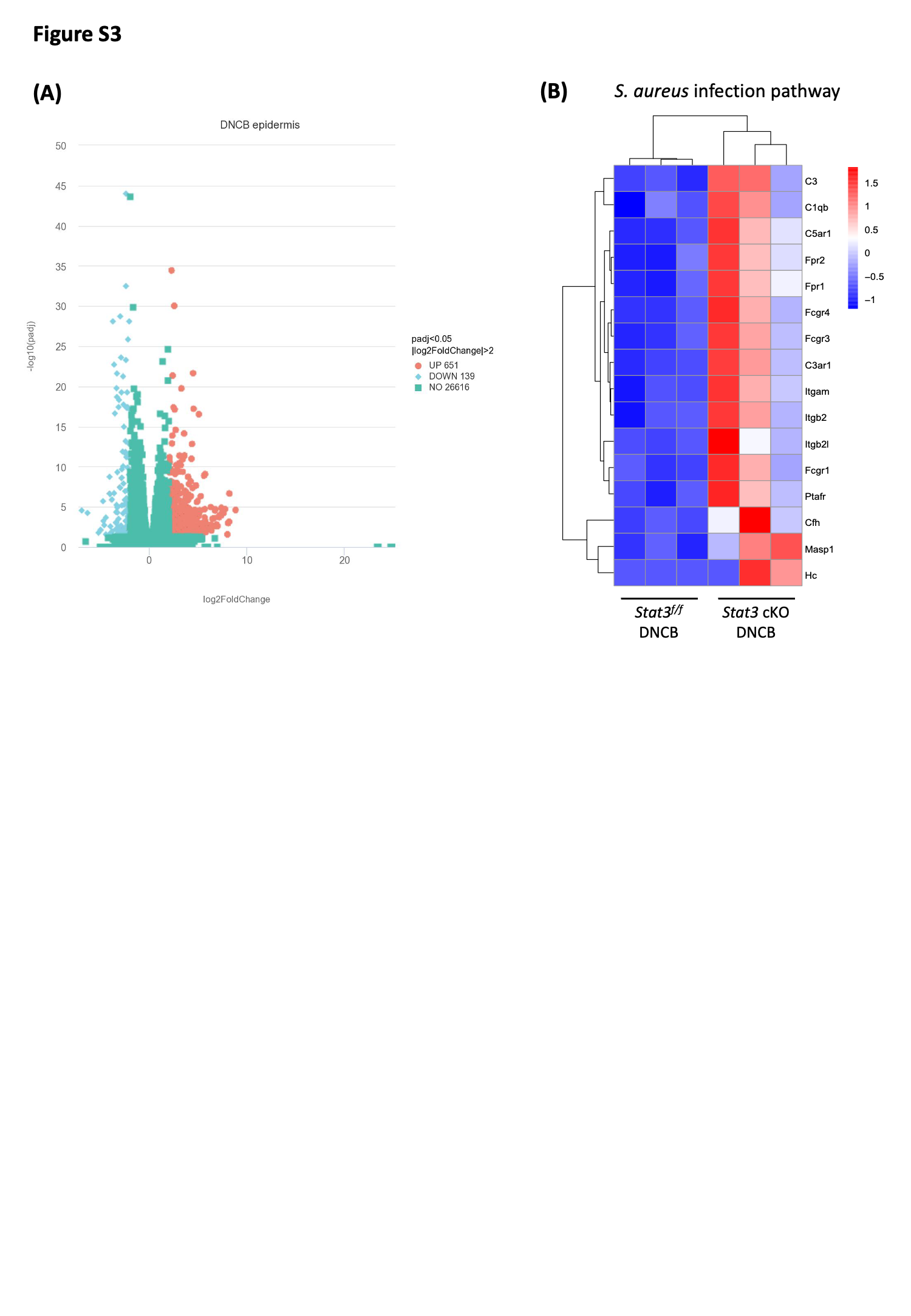

Supplement: Supplementary file 3 [file Image_3.tiff]
