# Supplementary material for: Epidermal keratinocyte-specific STAT3 deficiency aggravated atopic dermatitis-like skin inflammation in mice through TSLP upregulation
Source: Front Immunol. 2023 Nov 20;14:1273182. doi: 10.3389/fimmu.2023.1273182 (PMC10694200; doi:10.3389/fimmu.2023.1273182)
Supplement: Supplementary file 4 [file Table_1.docx]

**SUPPLEMENTARY MATERIALS**

**Supplementary Materials and Methods**

***Western blotting analysis***

After sacrifice, the mouse ear was removed, cut into pieces, and incubated with 0.5% dispase (Gibco, Waltham, MA) in 4°C overnight to separate the epidermis and dermis. Then, the epidermis was grinded to powder in mortar and lysed with RIPA buffer containing phosphatase and protease inhibitors. Equal amounts of lysate proteins (20 μg) were separated on SDS-PAGE gels, transferred to NC membranes (Millipore, CA, USA), and incubated with primary antibodies specific for STAT3 (Cell Signaling Technology, 12640, 1:4000), p-STAT3 (Cell Signaling Technology, 9145, 1:2000), and β-actin (Cell Signaling Technology, 4970, 1:2000). Blots were then rinsed in TBST buffer and further incubated in HRP-linked anti-rabbit IgG secondary antibodies for 2 hours. Proteins were visualized using the ECL substrate (Thermo Fisher Scientific, USA) and the chemiluminescence imager (Bio-Rad, Hercules, CA, USA). All experiments were repeated in triplicate.

***RNA sequencing and bioinformatics analysis***

RNA sequencing was performed on the total epidermal RNA from mouse ear skin after being treated with DNCB or vehicle for 21 days. The RNA integrity was assessed using the RNA Nano 6000 Assay Kit of the Bioanalyzer 2100 system (Agilent Technologies, CA, USA). Total RNA per sample was used as input material for library construction. PCR products were purified with the AMPure XP system (Beckman Coulter, Beverly, USA) and library quality was assessed on the Agilent Bioanalyzer 2100 system. Then, the clustering of the index-coded samples was performed on a cBot Cluster Generation System using the TruSeq PE Cluster Kit v3-cBot-HS (Illumina, San Diego, CA, USA). After cluster generation, the libraries were sequenced on an Illumina Novaseq platform and 150 bp paired-end reads were generated. After quality control, the clean reads were aligned to the reference genome using Hisat2 v2.0.5 and mapped read counts were estimated by featureCounts v1.5.0-p3. Differentially expression analysis was conducted by the DESeq2 R package (1.20.0). The *P*-values were adjusted using the Benjamini & Hochberg method. Adjusted *P* < 0.05 and |log2 fold change| > 2 were set as the threshold to define statistical significance. KEGG pathway analysis of DEGs was implemented by the clusterProfiler R package. An adjusted *P* < 0.05 was considered significant. The above RNA sequencing and data analysis were performed by the Novogene Bioinformatics Technology Co. (Beijing, China).

**Supplementary Figures**

**Supplementary Figure S1.** **The induction and validation of epidermal keratinocyte-specific STAT3 deficiency.** (A) Schematic diagram of the experimental design of tamoxifen induction. (B) Western blotting analysis of STAT3 and p-STAT3 expression in the epidermis after tamoxifen injection on D11. (C) Representative IHC staining of p-STAT3 in the ear skin. Bar = 100 μm.

**Supplementary Figure S2. Phenotype changes and STAT3 activation of *Stat3^f/f^* mice and *Stat3* cKO mice during DNCB or vehicle challenges.** (A) These phenotype photographs were taken weekly from Day 0 to Day 21. (B) Representative IHC staining of p-STAT3 in the ear skin. Bar = 100 μm.

**Supplementary Figure S3. The transcriptional profiling of the epidermis from DNCB-treated *Stat3^f/f^* mice and *Stat3* cKO mice.** (A) The volcanoplot diagram for the differentially expressed genes identified by RNA sequencing (n = 3, adjusted *P* < 0.05, |log2-fold change| > 2). (B) The enriched differentially expressed genes in the KEGG-identified *S.aureus* infection pathway.
